# Performance characteristics of the 5‐ring GE Discovery MI PET/CT scanner using AAPM TG‐126 report

**DOI:** 10.1002/acm2.14315

**Published:** 2024-02-28

**Authors:** Refaat Y. AlMazrou, Shadei F. Alanazi, Mohammed H. Alzaid, Razan S. Al‐Fakhranee, Shanli Ding, Osama R. Mawlawi

**Affiliations:** ^1^ Biomedical Physics Department King Faisal Specialist Hospital and Research Centre Riyadh Saudi Arabia; ^2^ General Electric HealthCare Riyadh Saudi Arabia; ^3^ Department of Imaging Physics MD Anderson Cancer Centre Houston Texas USA

**Keywords:** AAPM TG‐126, acceptance testing, NEMA NU 2‐2012, PET/CT

## Abstract

**Aim:**

To report on the performance characteristics of the 5‐ring GE Discovery MI PET/CT systems using the AAPM TG‐126 report and compare these results to NEMA NU 2‐2012 where applicable.

**Materials and Methods:**

TG‐126 testing was performed on two GE 5‐Rings Discovery MI scanners. Tests performed included spatial resolution, PET/CT image‐registration accuracy, sensitivity, count rate performance, accuracy of corrections, image contrast, scatter/attenuation correction, and image uniformity. All acquired data were analyzed using scanner console or free software tools as described by TG‐126 and the results were then compared to published NEMA NU 2‐2012 values.

**Results:**

Both scanners gave similar resolution results for TG‐126 and NEMA NU 2‐2012 and were within manufacturer specifications. Image‐registration accuracy between PET and CT using our clinical protocol showed excellent results with values ≤1 mm. Sensitivity using TG‐126 was 19.43 cps/kBq while for NEMA the value was 20.73 cps/kBq. The peak noise‐equivalent counting rate was 2174 kcps at 63.1 kBq/mL and is not comparable to NEMA NU 2‐2012 due to differences in phantoms and methods used to measure and calculate this parameter. The accuracy of corrections for count losses for TG‐126 were expressed in SUV values and found to be within 10% of the expected SUV measurement of 1. Image contrast and scatter/attenuation correction using the TG‐126 method gave acceptable results. Image uniformity assessment resulted in values within the recommended ± 5% limits.

**Conclusion:**

These results show that the 5‐ring GE Discovery MI PET/CT scanner testing using TG‐126 is reproducible and has similar results to NEMA NU 2‐2012 tests where applicable. We hope these results start to form the basis to compare PET/CT systems using TG‐126.

## 1 INTRODUCTION

Several national and international associations recommend performing acceptance testing on newly acquired positron emission tomography/computed tomography (PET/CT) systems. An example of these recommendations are the reports issued by the International Atomic Energy Agency (IAEA),^1^ the National Electrical Manufacturers Association (NEMA)^2^, ^3^ and the European Association of Nuclear Medicine.^4^ Performing acceptance testing ensures that the system is meeting the manufacturer specifications with acceptable results and performance. These recommendations however require the use of special sophisticated software and costly phantoms. The American Association of Physicists in Medicine (AAPM) has published Task Group 126 (TG‐126) report late 2019 “PET/CT Acceptance Testing and Quality Assurance”.^5^ This report provides a standardized set of tests that can be easily implemented in a quality assurance (QA) program for various PET/CT system platforms from different manufacturers.^5^, ^6^ The report recommends using standard inexpensive phantoms along with simple methods for data acquisition and processing.

TG‐126 report includes all the NEMA NU‐2 2012 tests (or equivalent tests) with the addition of two procedures—a PET/CT image registration accuracy (added in NEMA NU‐2 2018) and an image uniformity test. Some of the TG‐126 tests represent simplified versions of the NEMA NU‐2 2012 tests such as image quality test which uses the American College of Radiology (ACR) phantom^7^ instead of that from the International Electromechanical Commission (IEC). It also replaced the scatter fraction, count losses and randoms measurements including its specific phantom and testing procedure with imaging a standard uniform cylindrical water phantom that is used for the count rate performance, the assessment of accuracy of corrections, as well as image uniformity. For the resolution test, TG‐126 uses similar point sources and configurations as NEMA NU 2‐2012 while for the sensitivity test, it uses a similar procedure and phantom to that of NEMA NU 2‐2012 albeit using a single sleeve only.^5^, ^6^ Given that the phantoms and analysis methods used for TG‐126 and NEMA NU 2‐2012 testing are different, the expected characterization results would be different. Previously Akyol et al.^8^ published the performance of the GE 710 PET scanner using the TG‐126 method; however as of today, there exists no published reports on the 5‐ring GE Discovery MI PET/CT scanner performance characterization using the TG‐126 report.

In this study, the AAPM TG‐126 (will refer to them as AAPM method) performance characteristics of 5‐Ring GE Discovery MI PET/CT Scanners are presented. These results are compared (where applicable) with the NEMA NU 2‐2012 published results^9^ of the same scanners. Data were acquired on two identical systems installed recently at King Faisal Specialist Hospital and Research Centre, Riyadh, Saudi Arabia. To our knowledge these results represent the first complete characterization of the GE Discovery MI 5 ring PET/CT system according to the AAPM method.

## 2 MATERIALS AND METHOD

A detailed description of the AAPM method tests can be found in previous publications.^5^, ^6^

### 2.1 PET spatial resolution

**FIGURE 1:**
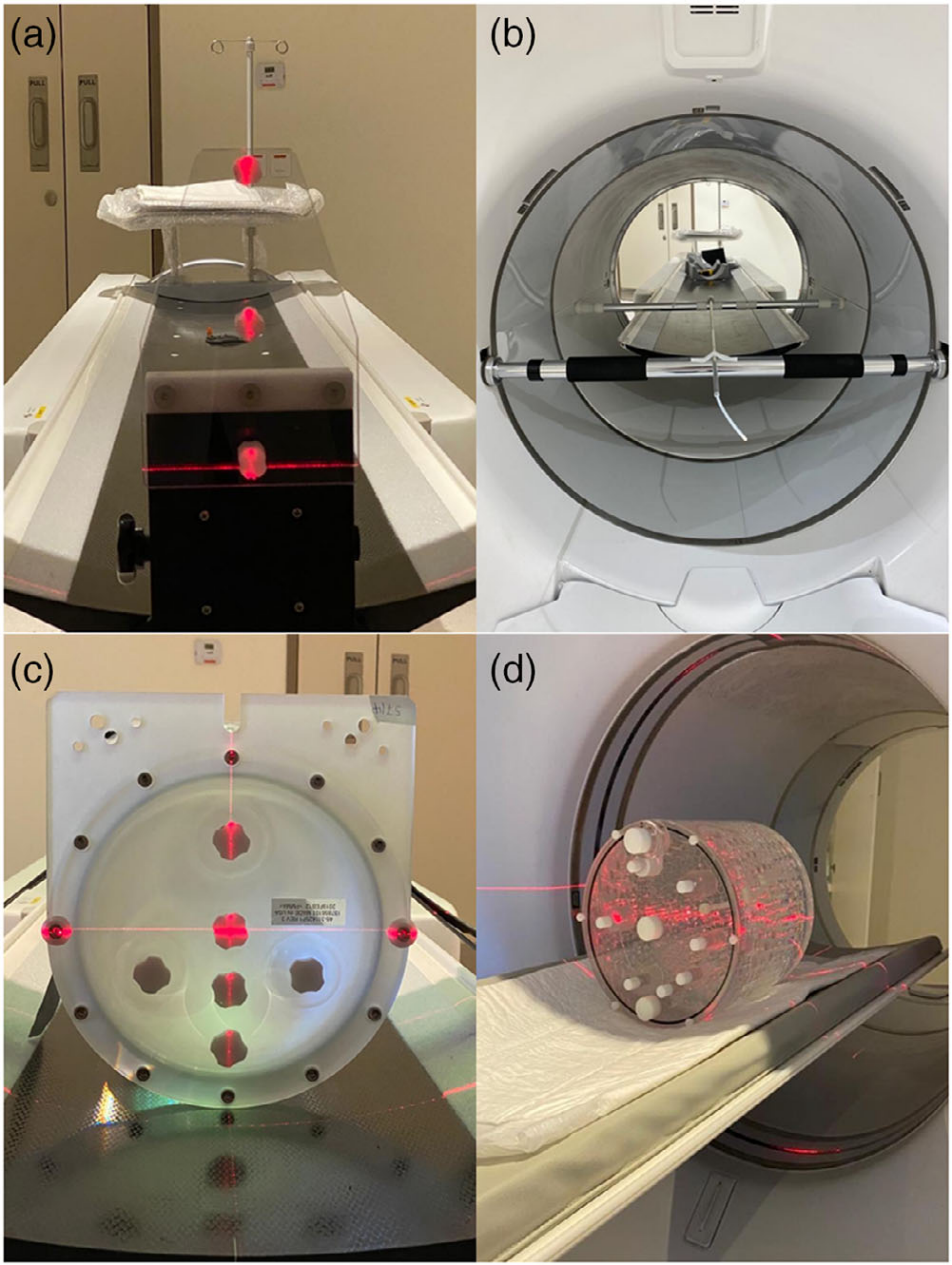
Experiment Setup for the various tests in the AAPM method showing (a) point sources for resolution measurement, (b) line source for sensitivity, (c) uniform cylindrical phantom for count rate performance, and (d) ACR phantom for image contrast and corrections evaluation.

Three point sources were made from a high concentration F‐18 activity (>185 MBq/mL) mixed with drops of CT contrast media. The point sources were then pulled into three 1.1 mm ID/75 mm long capillary tubes (Chase Scientific Glass Rockwood TN) using capillary action (each point source was <1 mm long) and positioned onto a source holder that came with the scanner (Figure 1a). The point sources were then positioned at 1, 10, and 20 cm along the y axis and imaged at two axial locations (center and 3/8 from the center of the axial field of view) according to the AAPM and NEMA NU 2‐2012 standards (Figure 1a).

For each axial position, PET data were acquired for 10 million counts and reconstructed using 384 × 384 matrix size, with 25 cm PET FOV (resulting in a pixel size of 0.65 mm) and no attenuation or scatter corrections applied. Both axial locations were reconstructed using 3 different algorithms—filtered back projection (FBP) with no image smoothing; an ordered subset expectation maximization (OSEM) iterative reconstruction (IR) without Time of flight (TOF) and resolution recovery (PSF) using 4 iterations and 34 subsets and 2 mm Gaussian smoothing (will refer to as VPHD); and our clinical protocol using a regularized IR algorithm with a penalization factor (*β*) of 350 using TOF and PSF (will refer to as QClear). The images were then exported to free software (ImageJ) and the radial, tangential, and axial FWHM values from line profiles through the point sources were measured for all the three reconstructions.

### 2.2 PET/CT image registration accuracy

The reconstructed images using the QClear technique from the previous test (PET Spatial Resolution) were used for this analysis. Axial fused PET and CT images were first displayed. Then for each point source (6 of them), image zoom was used to visually locate the center of the sources on the fused PET and CT images and to measure the distance between the centers of each source using the measuring tool available in the system.

### 2.3 Sensitivity

A simplified version of NEMA NU 2‐2012 sensitivity test using the fillable plastic tube (1 mm ID/3 mm OD) and the innermost sleeve (3.9 ID/6.4 mm OD and 700 mm long) of the NEMA NU 2‐2012 PET sensitivity phantom was utilized as described in the AAPM method. The tube was filled with 4.25 MBq (115 µCi) of F‐18 and imaged at two locations (0 and 10 cm off center). We used a slightly lower activity than the recommended AAPM range (5.55–7.4 MBq) in the line source due to the known higher sensitivity of this system and to ensure that we do not run into dead time issues as described in the AAPM method.

For each location, three separate one minute acquisitions were performed. The sleeve with line source where cantilevered between two tension rods that traversed the FOV of the scanner (Figure 1b). Images were reconstructed using 128 × 128 matrix size in 50 cm PET FOV resulting in a pixel size of 3.9 mm. The reconstruction was done using VPHD with 5 mm Gaussian filter and 34 subsets and 2 iterations. No attenuation correction was applied and the Z‐axis filter used was “Standard.”

For each reconstruction, the acquisition start time, the total prompt counts and the total random counts were retrieved from the DICOM image header using DICOM tags (0009, 106C), (0009, 1071), and (0009, 1072) respectively. The sensitivity was then calculated as described in the AAPM method and the average sensitivity for the three scans at each location was reported.

### 2.4 Count rate performance

A uniform cylindrical phantom (5640 mL) that usually comes with the PET/CT scanner was used for this test instead of the 70 cm long NEMA NU 2‐2012 scatter phantom. Two cylindrical phantoms were used, one for each scanner. The first scanner phantom was injected with about 525 MBq (14.2 mCi) of F‐18, while the second scanner phantom was injected with about 518 MBq (14.0 mCi). The phantoms were positioned on the patient couch and centered in the axial and transaxial FOVs (Figure 1C). We used a lower activity than the recommended AAPM range (700–750 MBq) in the phantom due to the known higher sensitivity of this system and to our prior knowledge that this activity would reach the desired max pseudo‐noise equivalent count rate (*R*_PNEC_).

A dynamic clinical protocol was used to acquire the data starting with a CT scan followed by multiple PET scans over a period of seven half‐lives (these are the baseline scans). PET scans were acquired for 15 min followed by 15 min delay. The dynamic scans were then reconstructed using our QClear clinical protocol with a FOV of 70 cm and a 256 × 256 matrix resulting in a pixel size of 2.73 mm.

For each image set of the dynamic series from each scanner, the *R*_PNEC_ was calculated according to Equation (1) below as described in the AAPM method^5^ and the results plotted versus the activity concentration.

(1) $$R_{PNEC}=\frac{{\left(R_{P}-R_{D}\right)}^{2}}{R_{P}+\left[\left(\frac{k}{2}-1\right)\times R_{D}\right]}$$

### 2.5 Accuracy of corrections

The reconstructed images of the uniform cylindrical phantom from the previous test (Count Rate Performance) were used in this test. For each reconstruction of the dynamic series, a circular region of interest (ROI) was drawn on the central slice (slice 45) occupying 75% of the inside diameter of the phantom. The SUV values were then computed for each ROI of each reconstruction of the dynamic series and plotted versus the activity concentration in the phantom.

### 2.6 Image contrast and scatter/attenuation correction evaluation

The AAPM method uses the American College of Radiology (ACR) procedure^7^ for this test. The patient dose in our facility is 370 MBq (10 mCi) with imaging set at an average of 60 min post administration, so the activity used for the hot cylinders was 13 MBq (351 µCi) injected in 1000 mL saline bag and the activity for the background was 30.3 MBq (820 µCi) as per the AAPM/ACR recommendations. The prepared phantom was used in both scanners and was positioned on the patient couch and centered in the axial and transaxial FOVs. A picture of this phantom and its position on the patient couch is shown in Figure 1d.

A whole body clinical protocol was used to image the phantom. Imaging started at about 60 min post phantom filling (55 min for scanner 1 and 65 min for scanner 2). The protocol parameters included 1 bed position of 2 min duration. The acquired PET data was then reconstructed using our clinical QClear protocol with FOV of 70 cm and 256 × 256 matrix resulting in a pixel size of 2.73 mm. The resultant images were re‐sliced to create 10 mm thick slices as required by the ACR.

The best slice showing the four hot cylinders was then selected for analyses as per the AAPM method to report on the SUV in the various cylinders of the ACR phantom.

### 2.7 PET uniformity assessment

Images from the “Count Rate Performance” test corresponding to an activity of 29.6 and 3.04 kBq/mL were used for this assessment. These activity concentrations are defined in the AAPM method and represent 1/2 to 1/3 the activity concentration at the *R*_PNEC_ and an activity concentration representing about 18.5 MBq (0.5 mCi) in the phantom.

On each image set and using the central slices, five circular ROIs with 30 mm diameter were drawn, at the center, 12, 3, 6, and 9 o'clock positions, with each ROI drawn 10 mm from the edge of the phantom. These five ROIs were copied to other slices covering the axial extent of the phantom, leaving three slices at the beginning and end of the phantom. We also analyzed the 10 mm thick re‐binned slices of the phantom where similar ROIs were drawn leaving off the two edge slices and the results were compared to the native reconstructed slices.

For each set of images (four sets), the integral uniformity within each slice ($IU_{N}$) and the integral axial uniformity ($IU_{AXIAL,M})$ were calculated according to Equations (2) and (3) below as described in the AAPM method.^5^, ^6^

(2) $$IU_{N}=\frac{\bar{\mathrm{RO}I_{\left(N,\max\right)}}-\bar{\mathrm{RO}I_{\left(N,\min\right)}}}{\bar{\mathrm{RO}I_{\left(N,\max\right)}}+\bar{\mathrm{RO}I_{\left(N,\min\right)}}}$$

(3) $$IU_{\mathrm{AXIAL},M}=\frac{\bar{\mathrm{RO}I_{\left(\max,M\right)}}-\bar{\mathrm{RO}I_{\left(\min,\;M\right)}}}{\bar{\mathrm{RO}I_{\left(\max,M\right)}}+\bar{\mathrm{RO}I_{\left(\min,M\right)}}}$$

## 3 RESULTS

**FIGURE 2:**
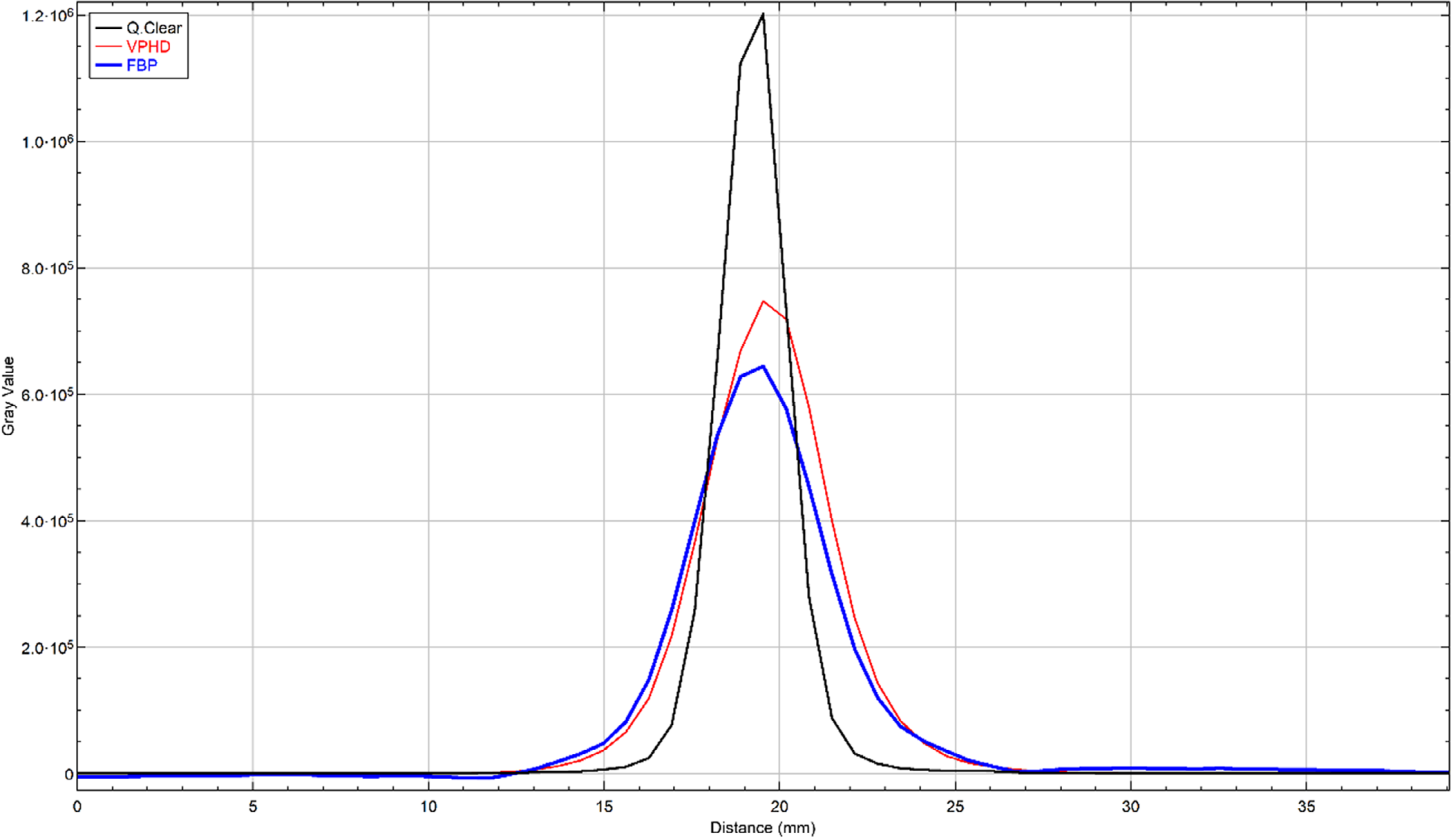
Line profiles of one of the point sources with the three different reconstructions algorithms.

We are reporting the average results from the two scanners for all the parameters measured where applicable. Table 1 shows the average resolution results (FWHM) at the two axial locations of the FOV for the three reconstruction algorithms (FBP, VPHD, and QClear) using both the AAPM method and published NEMA NU 2‐2012 results^9^ (where available). The results show that the AAPM and NEMA NU 2‐2012 methods have similar resolution values for FBP and VPHD at the various source locations with the latter reconstruction consistently showing better results than FBP while QClear had the best overall resolution measurements. Figure 2 shows line profiles of one of the point sources with the three different reconstructions.

**TABLE 1:** Average Spatial Resolution (FWHM in mm) at the two axial locations of the FOV for both scanners.

	AAPM FBP	NEMA FBP**^9^**	AAPM QClear using clinic. prot.	AAPM VPHD	NEMA VPHD**^9^**
	@ 1 cm radius				
Radial	4.99 (±0.30)	4.32 (±0.21)	2.48 (±0.42)	4.08 (±0.58)	3.73 (±0.06)
Tangential	4.79 (±0.45)	4.35 (±0.07)	2.71 (±0.48)	4.39 (±0.57)	3.91 (±0.09)
Axial	5.60 (±0.23)	5.05 (±0.06)	2.88 (±0.05)	4.57 (±0.15)	4.21 (±0.18)
	@ 10 cm radius				
Radial	5.72 (±0.13)	5.51 (±0.05)	2.68 (±0.32)	4.58 (±0.48)	4.73 (±0.06)
Tangential	5.38 (±0.63)	4.56 (±0.02)	2.58 (±0.42)	3.99 (±0.39)	3.86 (±0.03)
Axial	7.71 (±0.81)	6.49 (±0.35)	3.15 (±0.21)	4.75 (±0.19)	4.48 (±0.54)
	@ 20 cm radius				
Radial	7.52 (±0.25)	7.39 (±0.06)	3.43 (±0.36)	7.01 (±1.01)	7.15 (±0.01)
Tangential	5.17 (±0.44)	5.01 (±0.06)	2.84 (±0.48)	4.78 (±0.61)	4.29 (±0.26)
Axial	7.49 (±0.64)	6.56 (±0.33)	2.91 (±0.12)	4.56 (±0.10)	4.91 (±0.36)

Table 2 shows the results of the image registration test for both scanners at both positions (at the center and at the 3/8 of the axial FOV). These results show that the alignment of the PET and CT scanners for both systems are less than 1 mm which is within the AAPM limit of 1 PET pixel when using the clinical reconstruction algorithm. Table 3 on the other hand, shows the average sensitivity results for both AAPM method and published NEMA NU 2‐2012 results^9^ along with the percentage differences between them. These results show that the AAPM method gives results that are similar to NEMA NU 2‐2012 (19.43 ± 0.24 vs. 20.73 ± 1.04 cps/kBq) with an average difference of less than 10%. The AAPM method requires that annual testing should result in a sensitivity value within ± 5% of this baseline result.

**TABLE 2:** Average image registration test results for the axial fused PET and CT images in both positions.

	Average distance between PET and CT	
Axial position	Point source position	Distance (mm)
Center of FOV	(0,1)	0.55 (±0.15)
	(0,10)	0.85 (±0.05)
	(0,20)	0.90 (±0.10)
3/8 of FOV	(0,1)	0.65 (±0.15)
	(0,10)	0.65 (±0.15)
	(0,20)	0.85 (±0.15)

**TABLE 3:** Average sensitivity of both scanners using AAPM and NEMA NU 2‐2012 methods (cps/kBq) with their percentage differences.

Location	AAPM	NEMA**^9^**	Percentage difference
@ 0 cm	19.15 (±0.28)	20.84 (±1.13)	−8.1
@10 cm	19.71 (±0.21)	20.61 (±0.94)	−4.4
Average of both locations	19.43 (±0.24)	20.73 (±1.04)	−6.3

**FIGURE 3:**
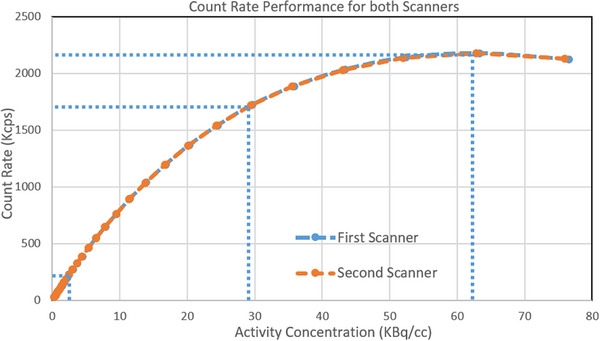
Pseudo‐noise equivalent count rate (*R*_PNEC_) versus activity concentration for both scanners showing the variation of *R*_PNEC_ with activity concentration. The dotted lines represent activity concentration at the *R*_PNEC_, half of the *R*_PNEC_ and at 3 KBq/mL corresponding to 18.5 MBq (about 0.5 mCi) in the phantom.

Figure 3 shows the count rate performance for the two scanners. The plots show the variation of count rate with activity concentration in the phantom as requested for the baseline measurement of count rate in the AAPM method. For annual testing we show three dotted lines representing activity concentration at the *R*_PNEC_, half of the *R*_PNEC_ and at 3 kBq/mL corresponding to 18.5 MBq (about 0.5 mCi) in the phantom as requested by AAPM for the analysis of the uniformity test. Table 4, on the other hand, shows the corresponding average pseudo‐peak noise equivalent count rates of these dotted lines with their average activity concentrations from both scanners using the AAPM method. Published NEMA NU 2‐2012 results^9^ are also shown in the table as a reference.

**TABLE 4:** Average pseudo‐peak noise equivalent count rates with their average activity concentrations from both scanners using the AAPM and NEMA NU 2‐2012 methods including these values for concentrations at half peak concentration and at activity of 0.5 mCi.

	AAPM @ peak *R*_PNEC_	AAPM @ half peak conc.	AAPM @ total act. of 0.5 mCi	NEMA @ peak NECR**^9^**
*R*_PNEC (K = 1)_ (Kcps)	2174.5 (±1.50)	1720.40 (±3.70)	270.75 (±0.35)	266.3 (±4.58)
Expected concentration (KBq/mL)	63.1 (±0.24)	29.6 (±0.1)	3.04 (±0.01)	20.8 (±0.48)

**FIGURE 4:**
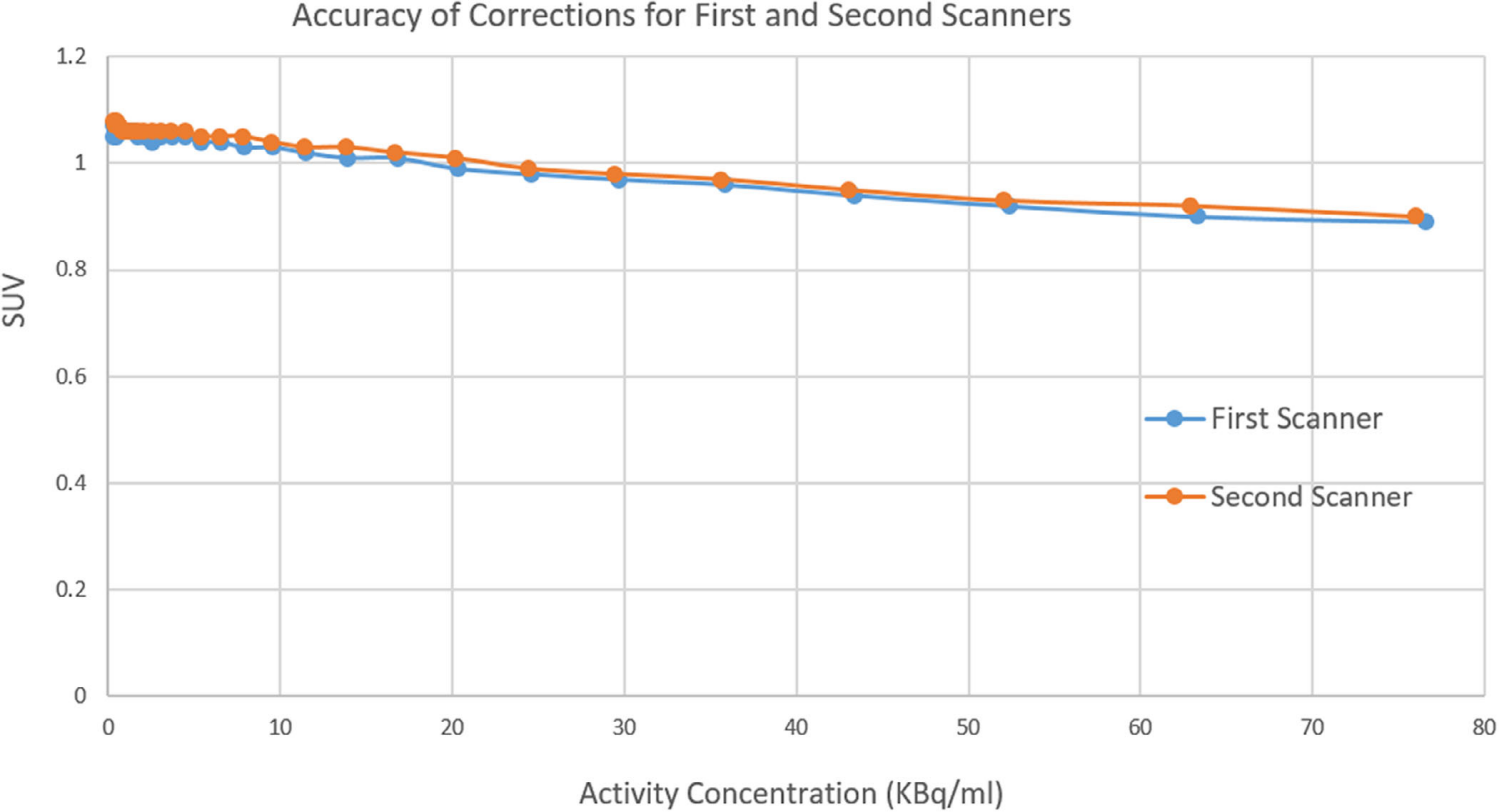
Accuracy of corrections for both scanners showing the variation of mean ROI SUV measurement with activity concentration. The expected SUV measurement should be equal to 1.

**FIGURE 5:**
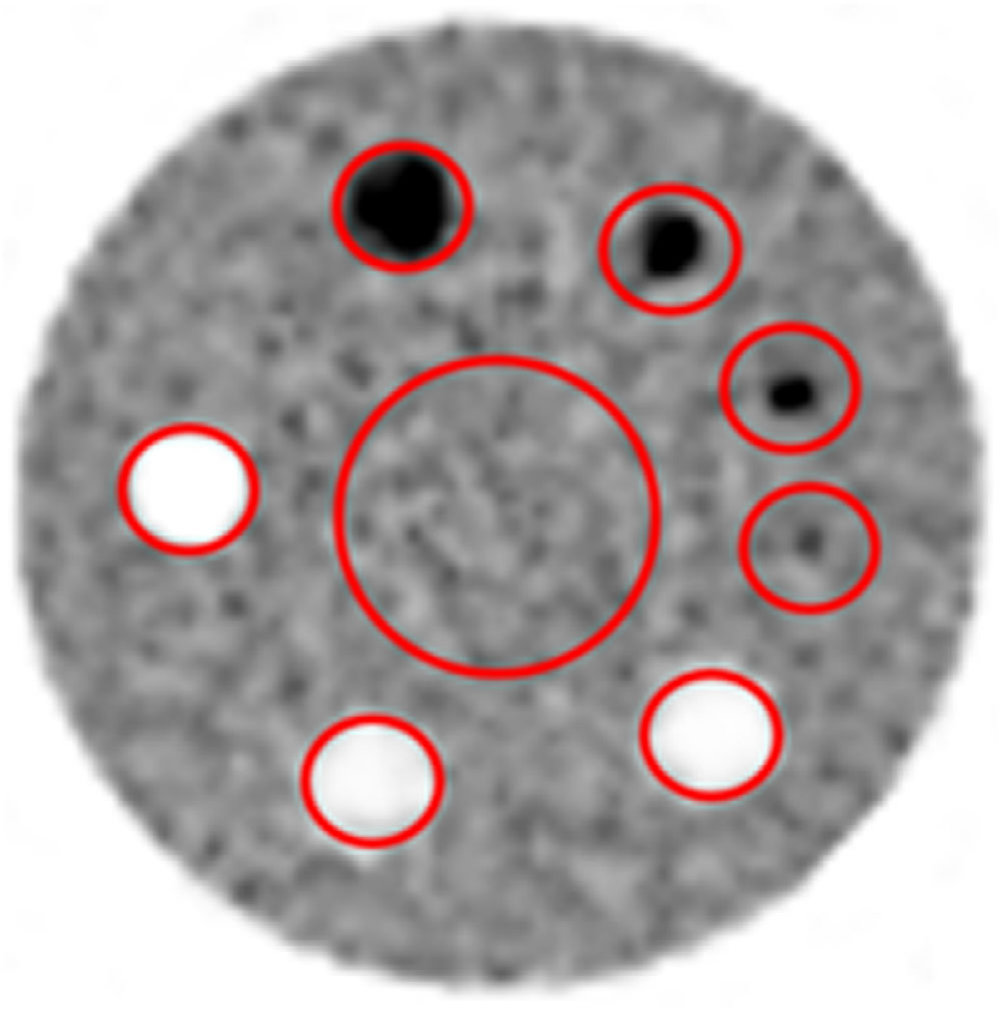
PET image of the ACR phantom showing a slice through the cylindrical inserts with various diameters to evaluate contrast and SUV measurement.

Figure 4 shows the results of the accuracy of corrections test for both scanners. The graph shows the variation of mean ROI SUV measurement with activity concentration from the count rate test for both scanners. The results show that the mean ROI SUV measurements for the various acquisitions of both scanners do not exceed the ± 10% limit defined by the AAPM method. Table 5 on the other hand, shows the image contrast and scatter/attenuation correction results (ACR Testing) for both scanners. A representative image of the ACR phantom is shown in Figure 5. All results are acceptable as per the ACR recommendations with the 12 mm cylinder and 9.5 mm rods visible at least with low contrast and with a mean background SUV of 0.995 (limit is 1 ± 15% [updated ACR data ± 12%]), maximum SUV in the 25 mm hot cylinder of 2.77 (limit is between 1.87 and 2.91), and the ratio of 16–25 mm hot cylinders of 0.873 (limit is >0.7).

**TABLE 5:** Average PET image contrast and scatter/attenuation correction results (ACR testing) of both scanners.

	**Background**	**Teflon**	**Air**	**“Cold” water**
**Mean SUV**	0.995 (± 0.005)	0.250 (± 0.000)	0.280 (± 0.020)	0.180 (± 0.000)
**Minimum SUV**		0.000 (± 0.000)	0.100 (± 0.020)	0.025 (± 0.005)
	**25** **mm “Hot”**	**16** **mm “Hot”**	**12** **mm “Hot”**	**8** **mm “Hot”**
**Maximum SUV**	2.770 (± 0.030)	2.430 (± 0.060)	2.395 (± 0.055)	1.905 (± 0.015)
**Ratio to background mean SUV**	2.784 (± 0.016)	2.443 (± 0.073)	2.407 (± 0.067)	1.915 (± 0.025)
**Ratio to 25** **mm “Hot” max SUV**	1.000 (± 0.000)	0.878 (± 0.031)	0.865 (± 0.029)	0.688 (± 0.013)

The Integral Uniformity within a slice ($IU_{N}$) and across Slices ($IU_{AXIAL,M})$ were measured at two activity concentrations of 29.6 and 3.04 kBq/mL (as described in the methods section) in the phantom using two different slice thicknesses (2.8 mm standard clinical image thickness, and 10 mm thick representing the ACR recommendations). Table 6 shows the average uniformity results of both scanners for both activity concentrations and slice thicknesses. The results show that the integral uniformity within the slice as well as across slices is less than 5% for both activity concentrations and slices thicknesses. These values are within the AAPM limits of 5%. As expected, both uniformity values were smaller for the thicker slices and higher activity concentration in the phantom.

**TABLE 6:** Average uniformity results of both scanners for both activity concentrations and slice thicknesses.

	2.8 mm thick slices		One cm thick slices	
	At 0.5 mCi	At half max	At 0.5 mCi	At half max
Activity conc. (KBq/mL)	3.04 (±0.01)	29.57 (±0.11)	3.04 (±0.01)	29.57 (±0.11)
Integ. unif. within slice (IU_I_) (%)	3.83 (±0.42)	3.03 (±0.51)	2.65 (±0.05)	1.85 (±0.07)
Integ. unif. across slices (IU_AXIAL_) (%)	4.53 (±0.43)	3.37 (±0.62)	2.35 (±0.05)	1.94 (±0.18)

## 4 DISCUSSION

This study evaluates the performance of a GE Discovery MI 25 cm PET/CT scanner using the AAPM method and compares these results to those from NEMA NU2‐2012. There is a newer version of the NEMA standard for PET systems—NU2‐2018. The difference between these two standards is limited to an assessment of the TOF timing resolution and a measurement of the registration accuracy between the PET and CT. Since the first of these two tests (TOF timing resolution) is not covered by the AAPM method while the second test is covered by the APPM method, comparing the AAPM results to either of the NEMA standards would result in the same outcome.

The scanner performance characterization using the AAPM method is based on analyzing reconstructed images (compared to the NEMA approach which uses raw data) without any preference to what reconstruction algorithm and associated parameters should be used. In this regard, this work makes use of our standard clinical image reconstruction protocol (QClear), previously described, to analyze images for the AAPM method.

The resolution testing for the AAPM and NEMA NU 2‐2012 methods are similar to one another and hence the results of these tests are expected to be similar as shown in Table 1 for both FBP and VPHD. There were no published results for QClear to include in this table. The resolution results for VHPD reconstruction are better than FBP given the well‐known effect of improved resolution of point sources in air when using IR techniques due to the nonlinear behavior of these algorithms along with their non‐negativity constraints.^10^ The same applies to the QClear clinical protocol that uses regularized reconstruction which further enhances the resolution results due to its inclusion of PSF and TOF during image reconstruction which further improves the signal to noise ratio of areas of interest by boosting the signal while suppressing the surrounding noise.^10^, ^11^

The results of image registration accuracy show that the distance between the centers of all point sources between the PET and CT images are less than 1 mm which is smaller than the size of a PET image pixel size when using the clinical protocol as required by the AAPM method. No comparison is made with NEMA NU2‐2012 since no such test was included in that publication. Our registration accuracy results however are within the manufacturer specifications (5 mm) defined as described in the NEMA NU2 −2018 publication.

The sensitivity results for the AAPM method are slightly lower than the NEMA NU 2‐2012 method with an average value of 19.43 ± 0.24 cps/kBq for both scanners and both locations compared to that for NEMA of 20.73 ± 1.04 cps/kBq, a difference of about 6.7%. This difference is expected and is due to the presence of the smallest sleeve (causing attenuation) in the AAPM method as compared to the NEMA NU 2‐2012 approach that extrapolates the sensitivity measurements to represent results without any attenuation. A direct comparison from the results of the smallest sleeve from the NEMA NU 2‐2012 approach using published results^9^ shows that these values are very similar to one another (19.43 vs. 19.45 or a difference of <0.1%).

The noise equivalent count rate curves of the two scanners using the AAPM method were identical to each other as seen in Figure 3. The AAPM method for this test is easy to perform and to calculate the required values. The only drawback is that this test (similar to the NEMA NU 2‐2012 method) takes a long time to acquire the necessary data for baseline testing. However, during the follow‐up testing (annual testing), only two scan time points are needed, one at an activity concentration equivalent to half the *R*_PNEC_ while the other is when the total activity in the phantom is 18.5 MBq (0.5 mCi) as defined by the AAPM method. Table 4 shows the results of this test for both methods with the AAPM approach giving much larger values than the NEMA NU 2‐2012 approach. This is primarily due to the lack of subtracting scatter in the AAPM approach as compared to the NEMA NU2‐2012 approach as described in Equations (1) and (2). Given that this test uses a different phantom and data analysis compared to NEMA NU 2‐2012, the results of the two methods are incomparable to one another as seen in Table 4.

Similar to the accuracy of corrections test for NEMA NU 2‐2012, the AAPM method uses the data from the count rate performance test to evaluate the correction accuracy with varying activity concentration. The results of this test using the AAPM method show that the SUV values for varying activity concentrations are within ± 10% of the expected SUV measurement of 1 up to the activity concentration corresponding to the peak pseudo‐NECR. This implies that the scanner produces accurate quantitative measurements over a wide range of activity concentrations. A closer look at the results show that the scanner slightly overestimates the activity concentration at very low activity concentration while underestimating these values at high activity concentration which is similar to NEMA NU 2‐2012 results for this test.^9^

The image contrast and scatter/attenuation correction test were performed using the ACR procedure and phantom. This test was used by the AAPM method to replace the 70 cm long phantom used in the NEMA image quality test. The results of this test for both scanners were within the recommended ACR limits. These results cannot be directly compared to the NEMA NU 2‐2012 image quality test due to differences in the requested outcome measures between the two tests.

Finally, the uniformity test results showed that all within slice and across slice integral uniformity values were within the recommended ± 5% limits. The AAPM method did not recommend to re‐bin the slices to 10 mm thick to reduce the variability in uniformity. Our results however showed that re‐binning the slices to 10 mm thick result in better uniformity measures as expected.

## 5 CONCLUSION

To our knowledge this is the first study that reports on the 5‐ring GE Discovery MI PET/CT system characterization using the AAPM TG‐126 report and compares these results to those from the NEMA NU 2‐2012 tests (where applicable). Due to the lack of manufacturer PET scanner performance characterization using TG‐126, we hope these results start to form the basis by which PET/CT systems can be compared to one another.

## AUTHOR CONTRIBUTIONS

Refaat Y. AlMazrou, Osama R. Mawlawi, and Shadei F. Alanazi contributed to the study conception and design; Refaat Y. AlMazrou, Shadei F. Alanazi, Mohammed H. Alzaid, and Razan S. Al‐Fakhranee participated in phantoms preparation, tests performance, and data analyses. The first draft of the manuscript was written by Refaat Y. AlMazrou, Osama R. Mawlawi, and Shadei F. Alanazi. Finally, Osama R. Mawlawi, Shanli Ding, Refaat Y. AlMazrou, and Shadei F. Alanazi reviewed, commented and finalized the manuscript.

## CONFLICT OF INTEREST STATEMENT

The authors declare no conflicts of interest.
